# Predicting the risk of relapsed or refractory in patients with diffuse large B-cell lymphoma via deep learning

**DOI:** 10.3389/fonc.2025.1480645

**Published:** 2025-03-03

**Authors:** Dongshen Ma, Yuqing Yuan, Xiaodan Miao, Ying Gu, Yubo Wang, Dan Luo, Meiting Fan, Xiaoli Shi, Shuxue Xi, Binbin Ji, Chenxi Xiang, Hui Liu

**Affiliations:** ^1^ Department of Pathology, The Affiliated Hospital of Xuzhou Medical University, Xuzhou, China; ^2^ Department of Sciences, Geneis Beijing Co., Ltd., Beijing, China; ^3^ Department of Pathology, Xuzhou Medical University, Xuzhou, China

**Keywords:** diffuse large B-cell lymphoma, histopathological images, clinical features, relapsed or refractory, deep learning

## Abstract

**Introduction:**

Diffuse large B-cell lymphoma (DLBCL) is the most common type of non-Hodgkin lymphoma (NHL) in humans, and it is a highly heterogeneous malignancy with a 40% to 50% risk of relapsed or refractory (R/R), leading to a poor prognosis. So early prediction of R/R risk is of great significance for adjusting treatments and improving the prognosis of patients.

**Methods:**

We collected clinical information and H&E images of 227 patients diagnosed with DLBCL in Xuzhou Medical University Affiliated Hospital from 2015 to 2018. Patients were then divided into R/R group and non-relapsed & non-refractory group based on clinical diagnosis, and the two groups were randomly assigned to the training set, validation set and test set in a ratio of 7:1:2. We developed a model to predict the R/R risk of patients based on clinical features utilizing the random forest algorithm. Additionally, a prediction model based on histopathological images was constructed using CLAM, a weakly supervised learning method after extracting image features with convolutional networks. To improve the prediction performance, we further integrated image features and clinical information for fusion modeling.

**Results:**

The average area under the ROC curve value of the fusion model was 0.71±0.07 in the validation dataset and 0.70±0.04 in the test dataset. This study proposed a novel method for predicting the R/R risk of DLBCL based on H&E images and clinical features.

**Discussion:**

For patients predicted to have high risk, follow-up monitoring can be intensified, and treatment plans can be adjusted promptly.

## 1 Introduction

Diffuse large B-cell lymphoma (DLBCL) is a highly heterogeneous malignancy, and the most common type of non-Hodgkin’s lymphoma (NHL) in humans (1, 2). Although the standard first-line treatment regimens such as R-CHOP (rituximab, cyclophosphamide, doxorubicin, vincristine, and prednisone) can cure 50% to 60% of patients, 40% to 50% of patients still experience primary refractory diseases or relapse in short period of time (3–8). Among patients with R/R disease, 15% to 25% experience disease progression during or after treatment (primary refractory disease), 20% to 30% relapse after achieving complete remission (CR), and approximately 5% experience disease progression after achieving partial remission (PR). Compared with non-relapsed and non-refractory patients, those with R/R disease have significantly worse prognoses (9). If the prognosis of patients can be assessed timely and accurately, and the treatment plan can be adjusted accordingly, the survival period of patients can be prolonged. Therefore, developing convenient and accurate prognostic evaluation methods for early risk prediction is of great significance in formulating personalized treatment strategies and improving patient prognosis. For high-risk patients, salvage treatments such as intensified induction therapy and autologous stem cell transplantation (ASCT) can be considered to improve treatment efficacy and patient survival rates (10, 11). On the other hand, after early detection of high-risk patients, more intensive monitoring and follow-up, as well as more aggressive therapeutic interventions, can be implemented, ensuring more rational and effective utilization of medical resources.

Nowadays, the prognosis of R/R DLBCL is far from reaching satisfaction even with salvage treatments (12). The main reason for patients developing R/R diseases is chemotherapy resistance to current R-CHOP treatment, which is associated with activated B-cell-like (ABC) cell-of-origin, aggressive genetic abnormalities, or an inhibitory tumor microenvironment (13). R/R DLBCLs often exhibit reduced sensitivity to a wide range of chemotherapy-based second-line regimens due to cross-resistance (14, 15), which also contributes to the poorer outcome of ASCT therapy (16, 17). Among patients receiving second-line intensive treatment, only 27% showed a response and received ASCT, with a median overall survival of only 10 months (18). Although patients who received salvage ASCT followed by chemotherapy could achieve a longer remission, the significant toxic effects of high-dose chemotherapy limited further treatment of elderly patients and those with complications (19). Furthermore, a multi-center study has demonstrated that the administration of rituximab prior to salvage therapy constitutes an adverse prognostic factor (20). If R/R patients are identified at an early stage and receive personalized treatment, such as proactive CAR-T cell therapy, it may provide a significant opportunity to improve the prognosis of this group (21). Therefore, early identification of potential R/R patients after diagnosis is crucial for selecting an intensive treatment regimen.

Currently, several biomarkers based on genetic aberrations or pathological factors have been utilized to predict the prognosis or risk of R/R disease, including *MYC/BCL2* rearrangement, cell-of-origin (COO) classification, or the international prognostic index (IPI) (13). It has been reported that more intensive regimens may be warranted in double-hit patients with higher stage and high IPI, which may lead to improved outcomes (22). Moreover, novel prognostic evaluation models have been studied considering more indicators such as PET-CT findings, liver function tests, serum albumin levels, and peripheral blood cell counts (23–25). However, there is currently no model for predicting the risk of R/R disease of DLBCL patients based on H&E pathological images, nor is there a model that combines clinical features, molecular pathological features, laboratory test results, and pathological images for predicting the prognosis of DLBCL. Hematoxylin and eosin (H&E) staining is one of the commonly used staining techniques in pathology. It has been reported that nuclear morphological features extracted from H&E images provided valuable prognostic information in various malignancies (26, 27). With the advancement of artificial intelligence in the field of image recognition, computer-aided diagnosis and prognosis prediction based on ultrasound images, CT images, and H&E images have been implemented in the medical field (28). In particular, because the analysis of pathological findings only utilizes retrospective information generated during routine diagnosis, it has the advantages of being timesaving, non-invasive, and low-cost (29).

In this study, we proposed a combined deep learning model to predict the R/R risk of DLBCL patients based on clinicopathological features, laboratory test results, and whole slide H&E images. The average area under the ROC curve (AUROC) of the fusion model reached 0.71 ± 0.07 in the validation dataset and the mean AUROC was 0.70 ± 0.04 in the test set.

## 2 Materials and methods

### 2.1 Clinical data collection and preparation

We collected clinical information and H&E stained whole slide images (WSI) of patients diagnosed with DLBCL in Affiliated Hospital of Xuzhou Medical University from 2015 to 2018. All patients’ diagnoses complied with the 2016 revision of the World Health Organization classification of lymphoid neoplasms (30).

The inclusion criteria were defined as follows: patients with a confirmed diagnosis of DLBCL through biopsy; patients had complete clinical and follow-up information, as well as histological specimens. Exclusion criteria included: Cases involving transformation of other lymphoma types into DLBCL; Consultation cases, puncture specimens, etc. without wax blocks or with insufficient remaining wax block tissue, as well as cases with insufficient tumor cell content; patients with incomplete clinical information or with follow-up duration less than 36 months after completion of treatment; patients with other malignant tumors; patients with severe cognitive impairment, communication disorders, or mental illnesses. All patients underwent pathological examination, with H&E samples sourced exclusively from surgical biopsies. The detailed de-identified clinical information and H&E images were transferred to the investigators. The follow-up time of DLBCL patients in the TCGA database is relatively short and the clinical information is not comprehensive enough to determine the R/R status, so the data in the TCGA database is not included in this study.

In selecting clinical variables, we initially extracted clinical information of anonymized patients from the hospital system. Subsequently, we incorporated specific domain knowledge and insights from experts in this field, collaborating with lymphoma specialists to determine the variables to be included. This process primarily involved excluding outcome variables such as treatment response, time to first progression, and recurrence status. Furthermore, we converted some continuous variables into categorical variables based on clinical significance, for instance, converting the numerical value of white blood cell count into categories of normal, decreased, and increased white blood cell count. This ensured that our variable selection was not only statistically reasonable but also biologically or practically meaningful. Next, variables with missing values exceeding one-third were excluded. For categorical variables with missing values below one-third, we used the mode for imputation, while for continuous variables with missing values below one-third, the median was employed. Multicategory variables were subjected to One-Hot Encoding for processing.

### 2.2 Image acquisition and preprocessing

For patients enrolled in this study, we first collected high-resolution images (20X) of the H&E stained WSI. Since each tissue sample is prepared on a pathological glass slide, the WSI consists of tissue and white background, which is the glass surface of the pathology slide. Therefore, it is essential to automatically identify the tissue area in the WSI. For each WSI, RGB was converted to HSV to facilitate division of the tissue area and white background according to the specified color threshold (threshold=35) and unnecessary white backgrounds were not included in further processing (31). To ensure the predicted effect, we performed image smoothing on the binarization mask of the tissue region: 1). The median value of gray scale was used for filtering, which can not only remove noise but also effectively retain the edge information of the tissue, to relatively reduce the blur degree of the image. 2). Morphological opening operation was performed to remove small gaps and holes by first etching and then expanding. After that, we split the tissue area into small patches of 512 × 512 pixels and stored the coordinates of patches and WSI metadata information in hdf5 format.

We used convolutional networks to extract low-dimensional and high-order semantic features of each patch. Then, we extracted pathological features from the first 3 residual blocks of ImageNet pre-trained ResNet18 and each patch was mapped to a 1x256 feature vector (32).

### 2.3 Definitions

We divided the patients into two groups according to their response to treatment: 118 patients with R/R DLBCL were marked as 1, and 109 patients with non-relapsed & non-refractory DLBCL were marked as 0. Patients with relapsed DLBCL were defined as those who achieved CR after 2 or more cycles of first-line chemotherapy and relapsed after at least one month of discontinuation after 6-8 cycles of treatment. Patients with refractory DLBCL refer to those who have not achieved remission at any time during treatment, or whose disease progression is less than 1 month after achieving PR during the treatment process, or who have relapsed within 1 month after achieving CR (33).

### 2.4 Model construction

The dataset was divided into 70% training set, 10% validation set, and 20% testing set. The training set was used for neural network model fitting, and the test set was used to evaluate the final model. We conducted 10 repeat trials to minimize random errors.

In the process of modeling based on images, to obtain slide-level features, our method is similar to CLAM - a weak supervised method based on deep learning, which uses attention-based learning to automatically identify sub-regions with high diagnostic value, in order to accurately classify the whole slide (32, 34–36). CLAM is based on a multi-instance learning (MIL) framework-a weak supervised learning task and framework. It regards each WSI (called a “bag”) as composed of many (up to hundreds of thousands) smaller areas or patches (called “instances”). In our study, each patient has a WSI, given a bag $\text{X = }\{x_{1},\cdots ,x_{m}\}\;\in \;R^{M\times 256}$ represent patient data, which contains 256-dimensional instances (patches). We first compress each 256-dimensional patch-level through two MLP—the MLP includes a fully connected layer, a ReLU layer, and a dropout layer— to a 128-dimensional vector $h_{i}\;\in \;R^{1\times 128}(i=1,\cdots ,M)$ . The first two layers of the attention network $U_{a}\in R^{64\times 128}$ and $V_{a}\in R^{64\times 128}$ collectively are considered as the attention backbone shared by all classes, and $W_{a}\in R^{1\times 64}$ is the weight parameter of the attention module, the attention score *A_i_* of the *i^th^* patch is given by $A_{i}=\frac{\exp\{W_{a}(\tanh(V_{a}h_{l}^{T})⊙sigm(U_{a}h_{l}^{T}))\}}{\sum_{j=1}^{M}\left(\exp\{W_{a}(\tanh(V_{a}h_{j}^{T})⊙sigm(U_{a}h_{j}^{T}))\}\right)}$ . Then, the slide-level feature $\text{H }\epsilon \;R^{1\times 128}$ is given by $\text{H }=\;\sum_{i=1}^{M}A_{i}h_{i}$ .

While in the process of constructing prediction models based on clinicopathologic variables, we applied the feature importance attribute of random forest (RF) to select the important features including age, gender, tumor stage, COO, LDH, et al. (37, 38). Then, we chose features that have scores to train the model.

**Figure 1:**
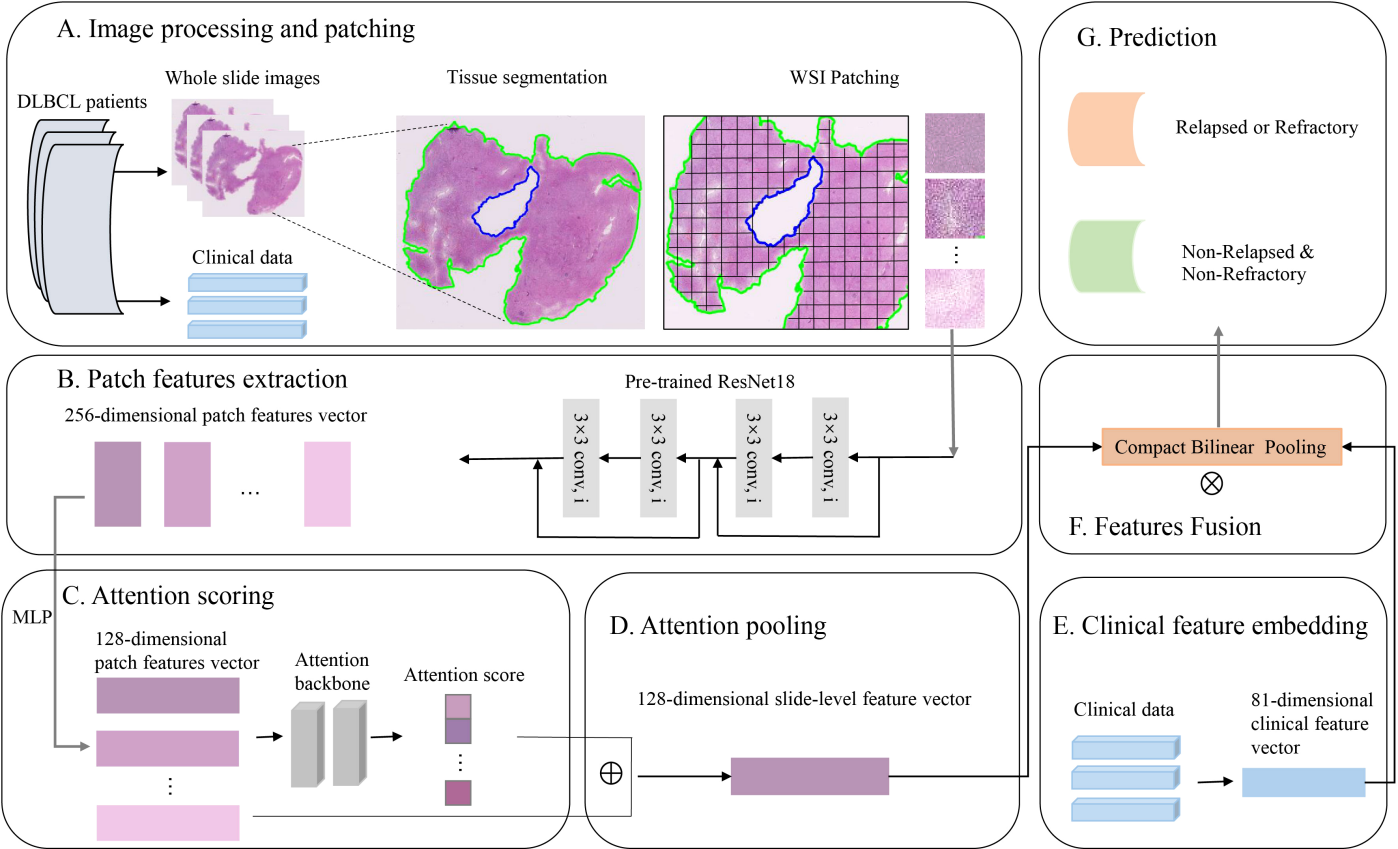
The process of fusion modeling. **(A)** H&E images preprocessing and patching. **(B)** Patch feature extraction using convolutional networks. **(C, D)** Attention scoring and pooling of images. **(E)** Clinicopathologic feature extraction and embedding. **(F)** Features fusion using compact bilinear pooling. **(G)** Construct a deep learning model to predict the relapsed or refractory status of patients with diffuse large B-cell lymphoma.

In the process of fusion modeling, we used the compact bilinear pooling (CBP) (39) method to fusion image features and clinicopathologic features. The clinicopathologic features were put into a multilayer perceptron (MLP) to embed vector and then were fused with image features using CBP. The fused features were fed into the final classification layer to obtain the final prediction result. The whole process of fusion modeling was shown in **Figure 1**.

### 2.5 Model interpretability

To explain the importance of different tissue regions in predicting the final slide level, we used an attention mechanism to calculate and save the attention scores of all patches extracted from the slides. These attention scores were normalized between 0 and 1 from low to high attention. These normalized scores were converted into RGB color using the divergent color map, and mapped to the corresponding position in the original pathological image according to the coordinate for visual recognition and interpretation. The high attention areas displayed in red (which contribute significantly to model prediction compared to other patches) and the low attention areas displayed in blue (which contribute less to model prediction compared to other patches).

To quantify the distribution of the cell densities across cohort, we used a Hover Net model, which was trained on the PanNuke dataset (a dataset containing 19 different types of cancer tissues), to perform nuclear segmentation annotation on five types of cells in WSIs, containing Neoplastic, Inflammatory, Connective, Dead, and Non-Neoplastic Epithelial cells. Then we calculated the cell density of these H&E images (40).

### 2.6 Statistical analysis

We used the “sklearn” library for python to compute metrics such as AUROC (41), accuracy, recall and F1 score for quantitatively evaluating the performance of the classifier. In order to deeply investigate the influence of clinicopathological characteristics on patients’ survival, we used the “survival” and “survminer” packages in R to conduct analyses. The survival outcomes between subgroups with different clinicopathological characteristics were assessed by Kaplan-Meier (KM) survival analysis and log-rank test.

## 3 Results

### 3.1 Baseline characteristics of the DLBCL patients in this study

In this study, we collected 227 patients diagnosed with DLBCL in Affiliated Hospital of Xuzhou Medical University from 2015 to 2018 with median follow-up 45.5 month. All patients had complete follow-up information and H&E pathological images (**Table 1**). All patients received chemotherapy, and none of the patients received hematopoietic cell transplantation as second-line therapy. Among them, 53.74% of patients were male, and 52.87% of the patients were older than 60 years old. 59.91% of the patients were in stage I-II when diagnosed. The chemotherapy received by the patients included CHOP-like (57.27%), CVAD-like (7.05%), MTX based (4.41%) or EPOCH-like (20.70%) regimens and 45.37% of the patients received rituximab during first-line treatment, and 44.05% of the patients achieved remission, including 26.43% showed CR and 17.62% showed PR. 80.18% of the patients had 1 or more extranodal extension sites, and 40.97% of patients had a survival period of less than 3 years. Among these DLBCL patients, 118 cases (51.98%) were determined to be R/R according to the guidelines for the diagnosis and treatment of DLBCL, among which, 47 were refractory (20.70%) and 71 were relapsed (31.3%).

**Table 1:** Basic information of DLBCL patients.

Clinicopathological variables	Category	Total cohort (227)	R/R Cases (118)	non-relapsed & non-refractory Cases (109)	*P*-value
Gender	Male	122 (53.74%)	68	54	0.1960
	Female	105 (46.26%)	50	55	
Age	<60	107 (47.13%)	49	58	0.1033
	≥60	120 (52.87%)	69	51	
Tumor stage	I	66 (29.07%)	28	38	0.0151
	II	70(30.84%)	31	39	
	III	50 (22.03%)	31	19	
	IV	41 (18.06%)	28	13	
Take Rituximab	Yes	103 (45.37%)	44	59	0.0011
	No	120 (52.86%)	74	46	
	Unknown	4 (1.76%)	0	4	
Response	CR	60 (26.43%)	2	58	< 2.2e-16
	PR	40 (17.62%)	20	20	
	SD	23 (10.13%)	7	16	
	PD	100 (44.05%)	86	14	
	Unknown	4 (1.76%)	3	1	
Number of extranodal extension sites	0	45 (19.82%)	19	26	0.1946
	≥1	182 (80.18%)	99	83	
IPI score	0-2	149 (65.64%)	72	77	0.0942
	3-5	54 (23.79%)	35	19	
	Unknown	24 (10.57%)	11	13	
Cell-of-origin	GCB	132 (58.15%)	67	65	0.8425
	Non-GCB	93 (40.97%)	50	43	
	Unknown	2 (0.88%)	1	1	
*MYC/BCL-2* Double-expression	Yes	59 (25.99%)	40	19	0.0047
	No	168(74.01%)	78	90	
*MYC/BCL2* Double-hit	Yes	2 (0.96%)	1	1	0.9782
	No	206 (99.04%)	105	101	

CR, complete remission; PR, partial remission; SD, stable disease; PD, progressive disease; GCB, germinal center B-cell like.

### 3.2 The performance of the model based on clinical information is superior to that of using H&E images

**Figure 2:**
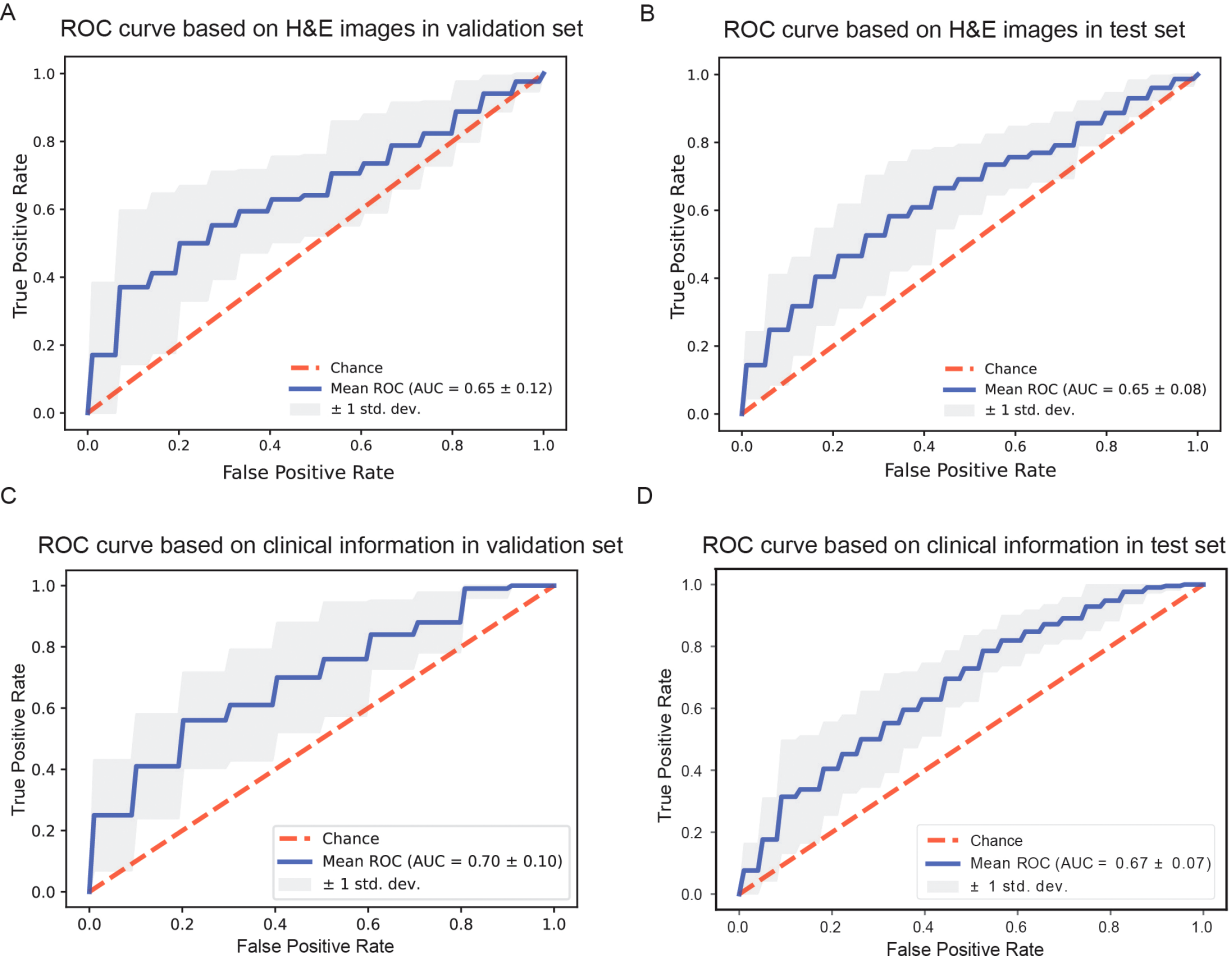
Comparison of model performance based on different information. **(A, B)** Receiver operating characteristic curves of models based on H&E stained whole slide images in the validation dataset and in the test dataset. **(C, D)** Receiver operating characteristic curves of models based on clinical information in the validation dataset and in the test dataset, respectively.

We marked R/R DLBCL patients as positive samples and non-relapsed & non-refractory DLBCL patients as negative samples, and then divided the two groups of patients into training sets, validation sets, and test sets at 7:1:2, respectively. During the H&E image modeling and clinical information modeling process, the patients used for model training were consistent. When we constructed model with H&E pathological images alone, the average AUROC of the model was 0.65 ± 0.12 (**Figure 2A**) in the validation dataset, and the average AUROC was 0.65 ± 0.08 in the test dataset (**Figure 2B**). We used the features importance attribute of RF to screen clinical and molecular pathological information and then selected features that have scores to construct a model using the RF algorithm. Finally, a total of 42 features were selected. After 10 rounds of modeling, in the validation dataset, the AUROC of the model was 0.70 ± 0.10 (**Figure 2C**), the average AUROC of the model was 0.67 ± 0.07 (**Figure 2D**) in the test dataset. As can be seen, the performance of the model based on clinical and molecular pathological information is superior to that using H&E images.

### 3.3 Some clinical features are strongly associated with relapsed or refractory of DLBCL

**Figure 3:**
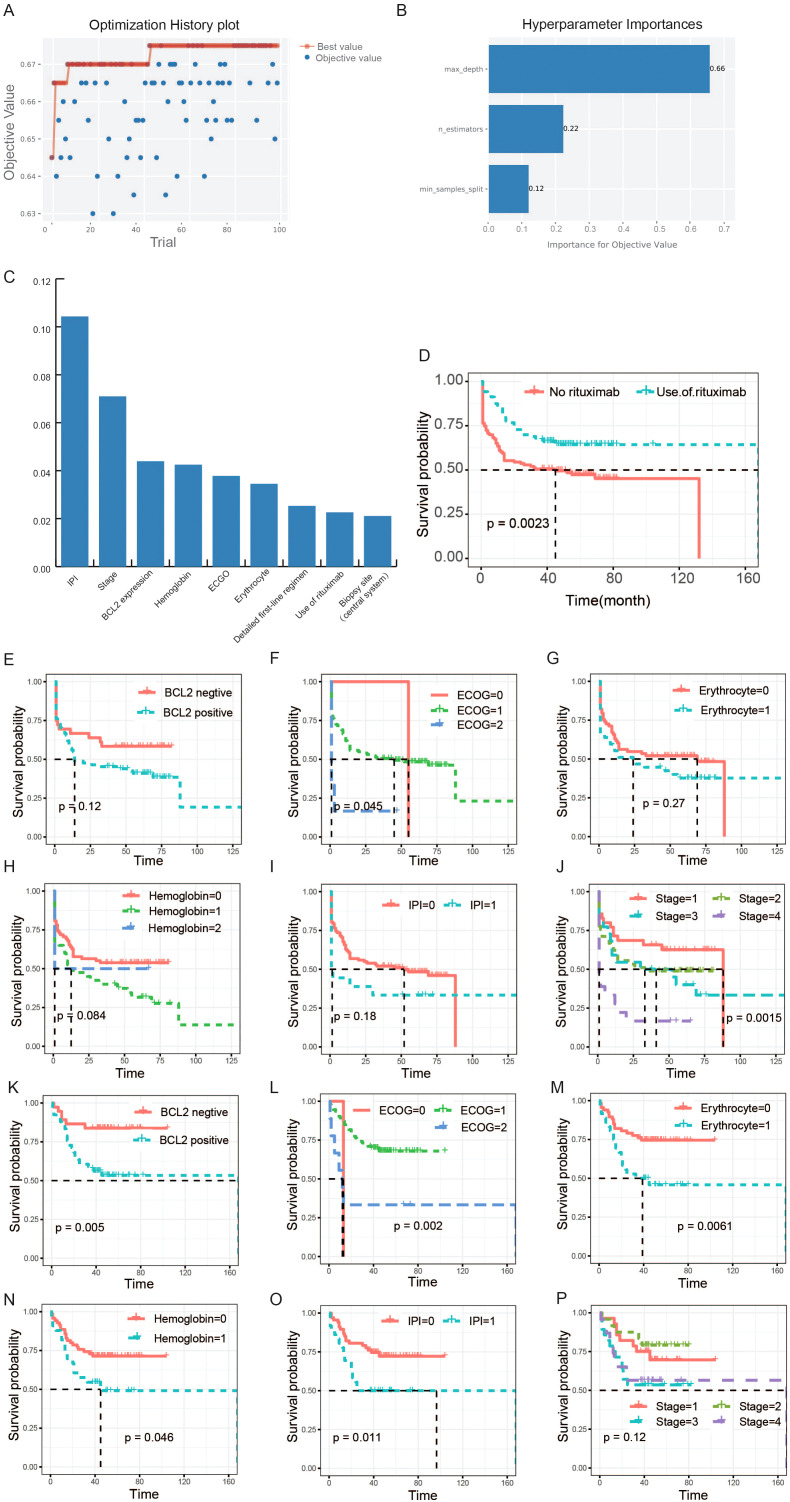
Selection of clinical features and their association analysis with survival. **(A)** The optimization history plot of modeling based on clinical and molecular pathological features. **(B)** Hyperparameter importance distribution in the optimal model. **(C)** The top nine features based on random-forest feature importance ranking. **(D)** The survival analysis of rituximab. The patients in this study were divided into two groups according to whether they were treated with rituximab. **(E-J)** The relationships between the six features and the prognosis of patients who did not receive rituximab treatment. **(K-P)** The relationships between the six features and the prognosis of patients who received rituximab treatment. The horizontal axis of the survival curve is the follow-up time (months), and the vertical axis is the survival rate.

During the process of building the prediction model using clinical and molecular pathological information, we employed the hyperparameter optimization framework ‘Optuna’ to optimize the three hyperparameters of RF: n_estimators、max_depth、min_samples_split, to improve the accuracy (ACC) of the model based on the validation set. After 100 iterations, we obtained the best combination of parameters: ‘n_estimators’: 80, ‘max_depth’: 9, ‘min_samples_split’: 6, and the corresponding ACC of the prediction model was 0.675. The specific optimization process and corresponding results of 100 experiments was shown in **Figure 3A**. In the process of optimizing hyperparameters, we found that max_depth was the most important hyperparameter, followed by min_samples_split, and finally n_estimators. The importance scores for objective value of each parameter were shown in **Figure 3B**. RF refers to a classifier that uses multiple decision trees to train and predict samples, and can analyze the importance of different features based on the contribution of each feature on each tree. The contribution metrics included the Gini index and out-of-bag data error rate. We employed the Gini index to evaluate the features. All features were sorted based on their scores and included in model training. In theory, the higher the ranking of features, the more closely related to the relapsed and refractory of DLBCL. We chose the top nine features to display in **Figure 3C**. Among these nine features, the utilization of rituximab is an important factor affecting the prognosis of DLBCL patients (P=0.0023, **Figure 3D**). So we divided the patients into two parts according to whether they were treated with rituximab. Then, we used survival analysis to further analyze the relationship between the top six features and the prognosis of patients. Specifically, patients were grouped based on eigenvalues and survival curves were used to describe the survival status of each group. The Kaplan-Meier curves of patients that did not receive rituximab treatment were shown in **Figures 3E–J**, and the others were displayed in **Figures 3K–P**. The Eastern Cooperative Oncology Group (ECOG) Score and the stage of disease were strongly correlated with the prognosis of patients with DLBCL (P <0.05) when they did not receive rituximab treatment. After receiving rituximab treatment, the stages was no longer significantly correlated with the prognosis of patients, indicating rituximab treatment changed the prognosis of patients. The cut-off value for positive expression of BCL-2 is 70% or more of tumor cells expressing BCL-2. As can be seen, the survival probability of the BCL-2 positive group was lower than BCL-2 negative group, and in the rituximab treated group, the difference was significant. Some patients with DLBCL may experience a decrease in erythrocyte count. According to the changes in erythrocyte count, patients were divided into two groups. The KM survival curve showed that the overall survival rate of the normal group was superior to the group with decreased erythrocyte count. According to whether the hemoglobin is abnormal, DLBCL patients were divided into three groups. The overall survival rate of the normal hemoglobin group was higher than that of the abnormal group. According to the international prognostic index (IPI) score, patients were divided into two groups: IPI scores of 0, 1, and 2 were marked as 0, while 3 and above were marked as 1. The overall survival rate of the 0 group was higher than the 1 group, whether they were treated with rituximab or not.

### 3.4 The visual analysis makes image-based modeling interpretable

**Figure 4:**
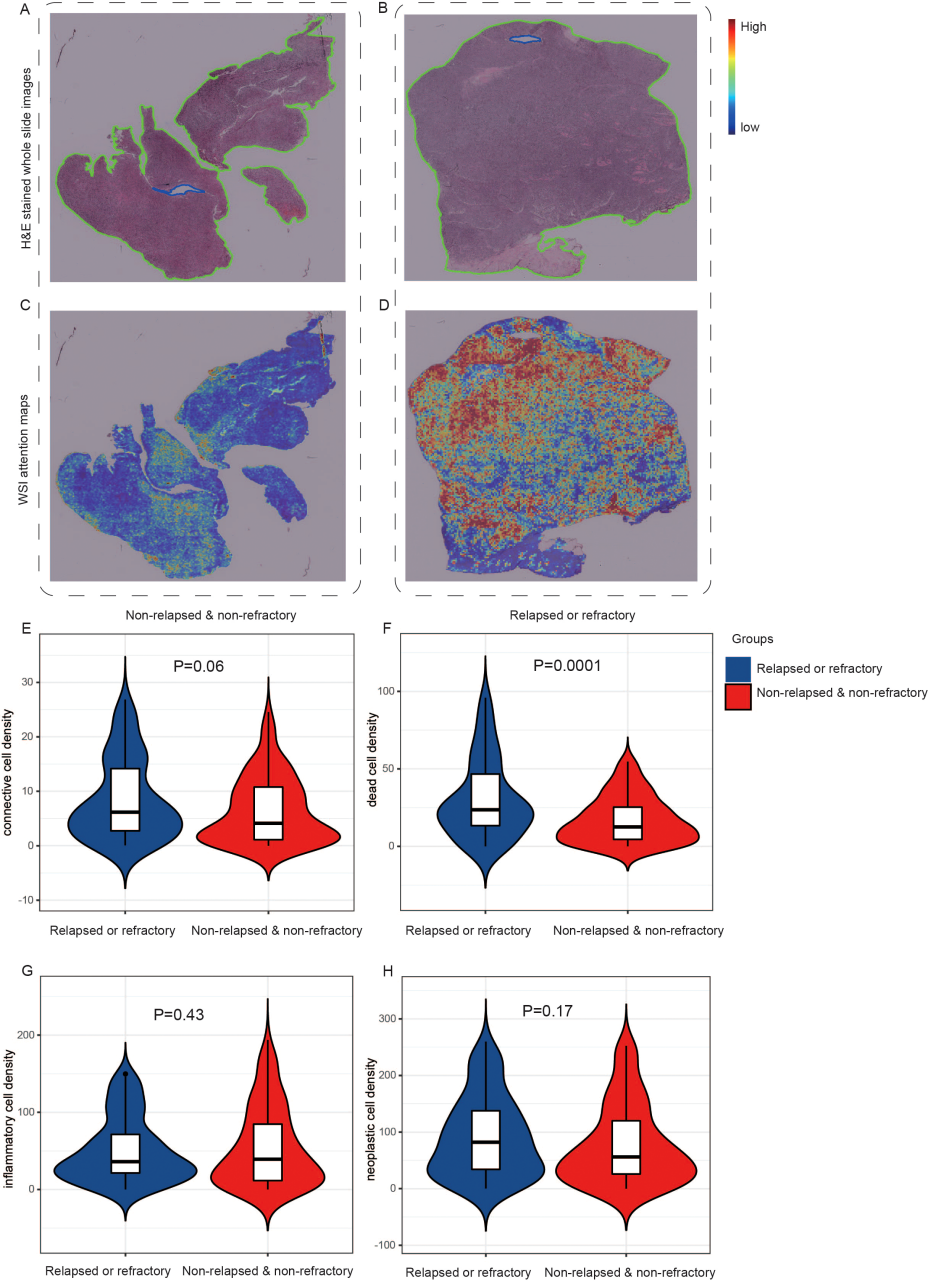
The visual analyses of H&E images-based modeling. One original H&E stained pathological image from the non-relapsed & non-refractory patient with DLBCL **(A)** and one image **(B)** from the relapsed or refractory patient with DLBCL. **(C, D)** The corresponding visualized attention heatmaps of the two H&E images, with red representing areas of high attention and blue representing areas of low attention. **(E-H)** The distribution of cell density in different queues. The inflammatory cell density in the R/R group was lower than that in the non-relapsed & non-refractory group, but there was no significant difference **(E)**. The density of connective tissue cells, dead cells, and neoplastic cells was higher in the R/R group **(F-H)**.

We used the attention mechanism to score each small patch of the H&E images, which were correctly classified using the H&E images-based model. The higher the score, the greater impact of the current patch on the patient’s final prediction. The red region meant a high attention area and the blue region represented a low attention area. Through the original H&E images and corresponding heatmap images, we obtained visual comparison graphs that can analyze which areas of the tissue were important to the prediction of relapsed or refractory. We chose H&E images and corresponding visualized images of two representative patients with different prognoses, which were shown in **Figure 4**. After reviewed by two pathology experts, they indicated that the original H&E stained pathological image from the non-relapsed & non-refractory patient with DLBCL (**Figure 4A**) revealed a low density of tumor cells and a rich background with pronounced fibrosis. However, the original H&E stained pathological image of R/R patient (**Figure 4B**) showed a high density of tumor cells and a relatively small number of background cells including T cells, histiocytes, and dendritic cells, with no significant fibrosis. Moreover, we can see the significant differences between these two groups of patients from the corresponding visualized heat-maps (**Figures 4C, D**).

Then, we further analyzed and demonstrated the distribution of cell density in different groups, as shown in **Figures 4E–H**. All tumorous cells are labeled as neoplastic, and other cells are labeled as non-neoplastic, including non-neoplastic epithelial, connective/soft tissue, inflammatory and dead cells. In our study, no cells were predicted to be non-neoplastic epithelial cell, so a total of four types of cell nuclei were predicted: neoplastic cells, connective/soft tissue, inflammatory and dead cells. In addition, we extracted 15 nuclear morphological features and used the average value to generate a 15 dimensional feature vector for each nuclei in the WSI. We compared all features between the two groups and listed them in the **Supplementary Table 1**. Then we used the SHAP package (42) to explain these features of the WSI and shown the mean SHAP value in **Supplementary Figure 1**. It was found that the inflammatory cell density in the R/R group was lower than that in the non-relapsed & non-refractory group, but there was no significant difference (**Figure 4E**). The density of connective tissue cells, dead cells, and neoplastic cells was higher in the R/R group (**Figures 4F-H**), with dead cell density significantly higher than in the non-relapsed & non-refractory group (P=0.0001). Inflammatory cells typically cannot become neoplastic, whereas connective tissue cells have the potential to become neoplastic (40).

### 3.5 Integrating H&E pathological images with clinicopathological information for modeling can improve the predictive performance

**Figure 5:**
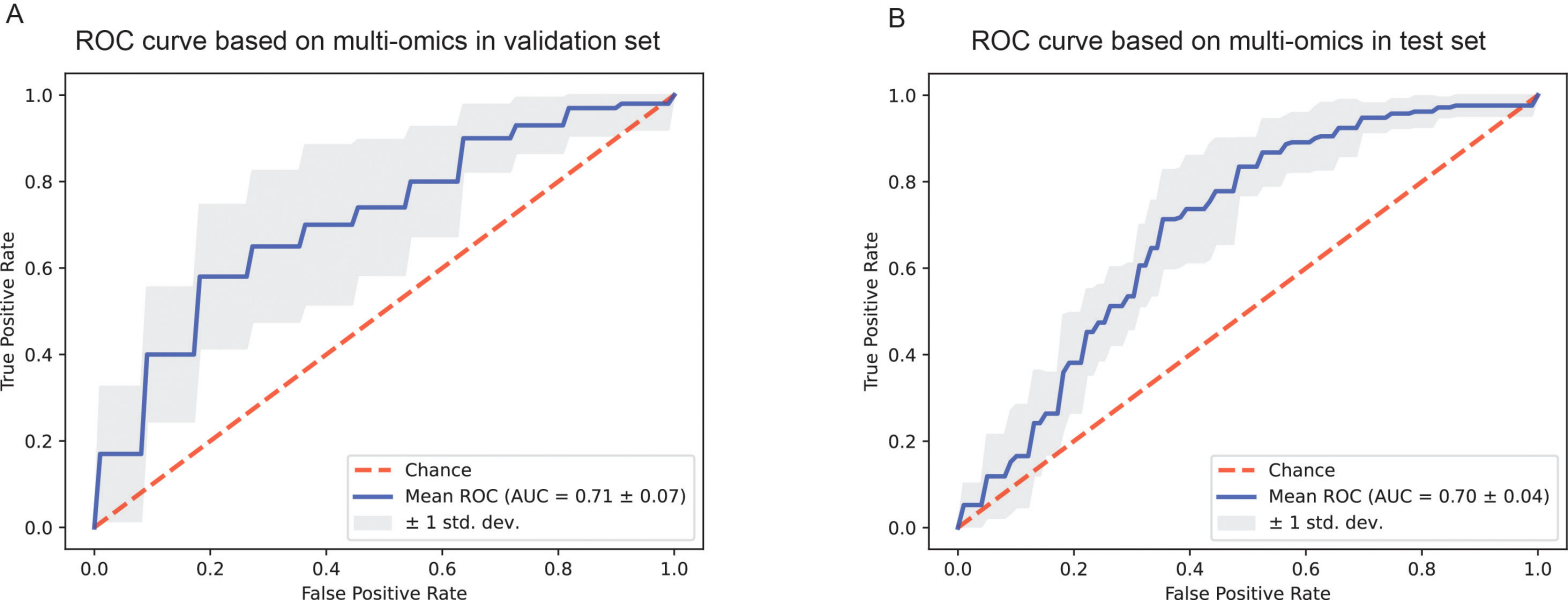
The ROC curves of the prediction model based on pathological images and clinicopathological information. **(A)** ROC curve of the combined deep learning model in the validation dataset. **(B)** ROC curve of the combined deep learning model in the test dataset.

Since the AUC values of the predictive models only based on H&E images and clinicopathological information respectively were lower than expected, we attempted to fuse H&E pathological image features and clinicopathological features using the CBP method to improve the performance of the prediction model. The grouping of patients and the partitioning of the dataset were the same as before. Finally, the mean AUC of the model in the validation set was 0.71 ± 0.07 after 10 rounds of repetition (**Figure 5A**), and the mean AUC in the test set was 0.70 ± 0.04 (**Figure 5B**). In addition, other performance parameters of the model were also improved, with the accuracy of the model was 0.658, the precision was 0.640, the recall was 0.643, and the F1 score was 0.629. While the accuracy of the model was 0.61, the precision was 0.60, the recall was 0.54, and the F1 score was 0.57 based on clinicopathological features, and the accuracy of the model was 0.50, the precision was 0.32, the recall was 0.38, and the F1 score was 0.32 based on pathological images. In addition, we employed four machine learning methods to construct classification model using the nuclear features extracted from H&E images. Furthermore, we combined the nuclear features, clinical features and H&E features obtained through the attention mechanism to develop a prediction model. The results were shown in **Supplementary Figure 2**.

## 4 Discussion

In this study, we collected clinical information, immunohistochemical results, laboratory test indicators and H&E stained pathological images of all DLBCL patients in Affiliated Hospital of Xuzhou Medical University from 2015 to 2018. After information matching and screening, there were 227 patients met the enrollment requirements. Then, we extracted pathological image features to predict R/R and non-relapsed & non-refractory DLBCL patients. To the best of our knowledge, this is the first study to construct a deep-learning predictive model utilizing H&E images to predict the prognosis of DLBCL patients. In addition, we sorted the clinical information, laboratory test indicators, and immunohistochemical information using the feature importance attribute of the random forest, and then constructed a predictive model with slightly better results than those based on pathological images alone. Finally, we developed a model that combining pathological image features with clinical information, laboratory indicators, and immunohistochemical indicators to predict R/R and non-relapsed & non-refractory risk of DLBCL patients, with an AUC of 0.71 ± 0.07 in the validation set and 0.70 ± 0.04 in the test set.

Hans et al. (43) classified DLBCL into germinal center B-cell like (GCB) and non-germinal center B-cell like (non GCB) subtypes based on the expression of CD10, bcl-6, and mum-1 in histopathological sections using immunohistochemistry. This classification could predict prognosis of patients, but the drawback is that the accuracy of classifying the same sample population is not high enough. It is far from enough to judge prognosis and select treatment based solely on COO classification during initial diagnosis. While the extant literature is sparse regarding the correlation between the COO and relapsed/refractory diffuse large B-cell lymphoma (R/R DLBCL). A recent study has elucidated that COO does not exhibit a significant association (*p*=0.285) with primary progressive disease which is characterized by either primary refractory disease, where the lymphoma does not respond to initial therapy, or relapsed disease occurring within 12 months post-treatment (44). It was reported that GCB cases accounted for 60.8% and non-GCB cases accounted for 39.2% of R/R DLBCLs (45), similar to our study. In our study, the R/R group included cases of primary refractory disease as well as all relapsed cases (relapsed after at least one month at the end of the treatment). Consistently, there was no significant correlation (*p*=0.84) between COO and the occurrence of R/R disease, which was shown in **Table 1**. Moreover, there was no significant correlation (*p*=0.27) between COO classification and overall survival of patients. In summary, our dataset is representative compared with other data.

We obtained a ranking of features related to relapsed or refractory risk of DLBCL patients using the feature importance attribute of RF. Through KM analysis, we found the use of rituximab and ECOG are strongly associated with survival rate. In previous studies, the expression of *MYC and BCL-2* had been proven associated with a poor prognosis in initially treated DLBCL and R/R DLBCL (46, 47). Most patients with refractory lymphoma manifest as double-hit lymphoma (*MYC-BCL2* rearrangement) (DHL) or double-expression lymphoma (*MYC-BCL2* overexpression) (DEL), with more invasive manifestations clinically. The prognosis of these patients is poor, and R-CHOP may not be the preferred treatment option. IPI, ECOG, and clinical stage of tumor also had been reported to have important influences in the outcome of DLBCL (10). However, there have been no reports on the correlation between hemoglobin content and the prognosis of DLBCL yet, it still need to be further studied. M. Detrait et al. collected data from 130 patients with DLBCL to construct a model for predicting primary refractory disease (48). They used a variety of variables, including demographic characteristics, clinical condition, disease characteristics, first-line therapy, and PET-CT scan realization after 2 cycles of treatment. The performance of our model surpasses three models in their study and is slightly inferior to two of them. Maybe because they incorporated the indicator of PET-CT scan realization after 2 cycles of treatment, which can provide an initial assessment of prognosis.

There are some limitations in this study. Firstly, the accuracy of predicting the risk of R/R and non-relapsed & non-refractory in DLBCL patients only based on pathological images is relatively low. We analyzed the possible reasons as follows: First, we used WSI instead of region of interest (ROI) for feature extraction, which may affect the performance. The results from Liu et al. illustrated the model based on WSI achieved the best predictive performance compared to tumor area and peri-tumor area in Lymph node metastasis status (49). In addition, it was concordant with previous studies that the biological information on oncological outcome was not limited to the tumor area (50). Moreover, the pathologist evaluated that the lymphoma cells spreading throughout the WSI, so we chose WSI as the input. Second, the number of collected samples was not large enough, so high heterogeneity combined with a small dataset may lead to poor performance of the training model. Third, extracting abstract features from H&E stained patches of DLBCL patients alone may not be a good predictor of patient prognosis, and this method is lack of interpretability. We may need to analyze different cell ratios and tissue components of the FFPE slides. Loi et al. reported that tumor infiltrating lymphocytes (TILs) were prognostic in triple negative breast cancer and predictive for trastuzumab benefit in early breast cancer (51). The interplay of sTILs, tumor cells, other microenvironment mediators, their spatial relationships, quantity, and other image-based features have yet to be determined exhaustively and systemically (52). The phenotypic information in pathological tissue reflected the overall effect of the tumor microenvironment on the behavior of cancer cells. Recent studies demonstrated that deep learning models can identify protein expression alterations and genetic mutations based on histological images across multiple cancer types (53, 54). So we assumed that we could predict the prognosis of DLBCL patients based on H&E stained pathological images. And, as can be seen from **Figure 4**, there were significant differences in H&E stained pathological images between R/R patients and non-relapsed & non-refractory patients after visualization.

Secondly, as the labels of R/R and non-relapsed & non-refractory are difficult to obtain, we only collected one valid dataset, there is still a lack of independent test datasets. We downloaded clinical pathological information and H&E images of DLBCL patients from TCGA, and after information matching, we found that the prognosis information is incomplete and could not be used as an independent test dataset.

In the future, we plan to optimize the research from the following aspects. Firstly, we will collect more data from our hospital and other hospitals to optimize the model. Secondly, we will continue trying different image preprocessing methods to improve the performance of the models, hoping provide a valuable reference for hematologists. Compared with traditional methods, artificial intelligence based image recognition and classification can extract more important information that cannot be recognized by human eyes. In addition, due to the reduction of differences in visual perception among different individuals, these high-dimensional features will also become more objective and reliable. We expect to construct a model with an optimal performance that combines clinical information, laboratory examination results, immunohistochemical indicators, and pathological images together. This model could predict the prognosis of DLBCL patients accurately in the early stage, and assist doctors in developing personalized treatment plans.

## Data Availability

The raw data supporting the conclusions of this article will be made available by the authors, without undue reservation.
