# Supplementary material for: Predicting the risk of relapsed or refractory in patients with diffuse large B-cell lymphoma via deep learning
Source: Front Oncol. 2025 Mar 3;15:1480645. doi: 10.3389/fonc.2025.1480645 (PMC11911189; doi:10.3389/fonc.2025.1480645)
Supplement: Supplementary file 1 [file DataSheet1.docx]

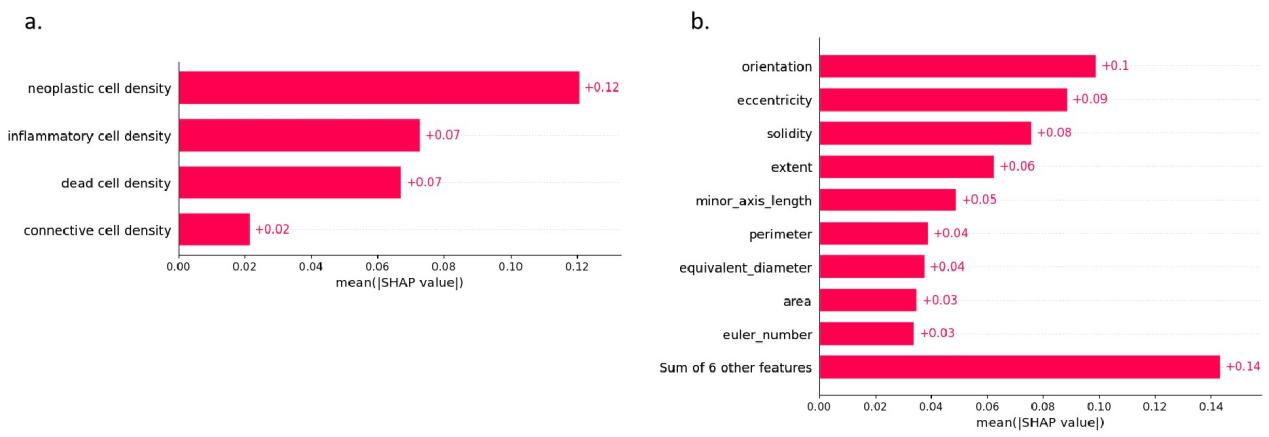


Supplementary Figure 1. The mean sharp value of all features. a. sharp values of cell densities. b. sharp values of nuclear morphological features.


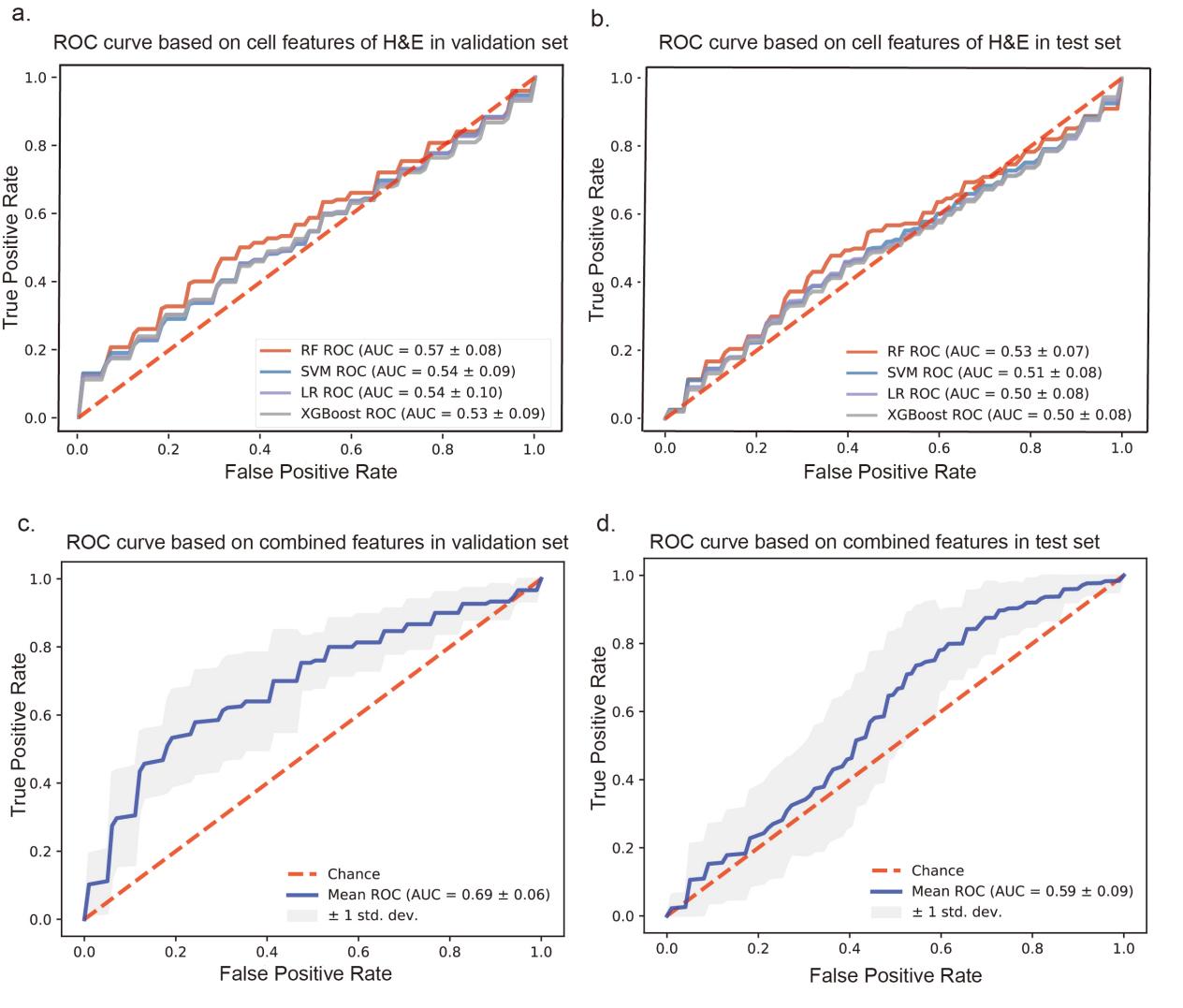


Supplementary Figure 2. The Comparison of model performances. (a-b) ROC curves based on cell features extracted using Hover-Net model. (c-d) ROC curves based on combined features, including cell features, clinical information, and patch features from H&E images extracted using convolutional networks.
