# Supplementary material for: Predicting the risk of relapsed or refractory in patients with diffuse large B-cell lymphoma via deep learning
Source: Front Oncol. 2025 Mar 3;15:1480645. doi: 10.3389/fonc.2025.1480645 (PMC11911189; doi:10.3389/fonc.2025.1480645)
Supplement: Supplementary file 2 [file Table1.docx]

In our study, a total of four types of cell nuclei were predicted: neoplastic cells, connective/soft tissue, inflammatory and dead cells. Assuming that different types of nuclear morphology are associated with relapsed or refractory states, we extracted 15 nuclear morphological features (Supplementary table 1).

Supplementary Table 1. Comparison of all features between the two groups

| **Feature** | **P value** |
| --- | --- |
| Connective cell density | 0.06 |
| Dead cell density | 0.0001 |
| Inflammatory cell density | 0.43 |
| Neoplastic cell density | 0.17 |
| eccentricity | 0.0017 |
| equivalent_diameter | <0.0001 |
| euler_number | <0.0001 |
| extent | 0.004 |
| area_filled | <0.0001 |
| inertia_tensor_eigvals_x | <0.0001 |
| inertia_tensor_eigvals_y | <0.0001 |
| major_axis_length | <0.0001 |
| minor_axis_length | <0.0001 |
| orientation | 0.0028 |
| perimeter | <0.0001 |
| solidity | <0.0001 |
| area | <0.0001 |
| bbox_area | <0.0001 |
| convex_area | <0.0001 |

Notes：

area: number of pixels in the region. bbox area: number of pixels of bounding box of the region. convex area: number of pixels of convex hull image, which is the smallest convex polygon that encloses the region. eccentricity: Ratio of the focal distance (distance between focal points) over the major axis length. equivalent diameter: the diameter of a circle with the same area as the region. euler number: number of objects in the region subtracted from the number of holes in those objects. extent: the proportion of pixels in the bounding box that are also in the region. filler_area: number of pixels of the region will all the holes filled in. inertia_ tensor_eigvals_x: the eigenvalues of the inertia tensor among x-axis in decreasing order. inertia_tensor_eigvals_y: the eigenvalues of the inertia tensor among y-axis in decreasing order. major_axis_length: the length of the major axis of the ellipse that has the same normalized second central moments as the region. minor_axis_length: the length of the minor axis of the ellipse that has the same normalized second central moments as the region. orientation: angle between the x-axis and the major axis of the ellipse that has the same second moments as the region. perimeter: perimeter of object which approximates the contour as a line through the centers of border pixels using a 4-connectivity. solidity: ratio of pixels in the region to pixels of the convex hull image.
